# 共价有机框架分子印迹聚合物复合材料的制备及其用于牛奶中痕量诺氟沙星的选择性富集

**DOI:** 10.3724/SP.J.1123.2021.03013

**Published:** 2022-01-08

**Authors:** Yang XIE, Yi ZHANG, Haizhu SHI, Zhaoju WU, Xuehong YU, Chungu ZHANG, Shun FENG

**Affiliations:** 西南交通大学生命科学与工程学院, 四川 成都 610031; School of Life Science and Engineering, Southwest Jiaotong University, Chengdu 610031, China; 西南交通大学生命科学与工程学院, 四川 成都 610031; School of Life Science and Engineering, Southwest Jiaotong University, Chengdu 610031, China; 西南交通大学生命科学与工程学院, 四川 成都 610031; School of Life Science and Engineering, Southwest Jiaotong University, Chengdu 610031, China; 西南交通大学生命科学与工程学院, 四川 成都 610031; School of Life Science and Engineering, Southwest Jiaotong University, Chengdu 610031, China; 西南交通大学生命科学与工程学院, 四川 成都 610031; School of Life Science and Engineering, Southwest Jiaotong University, Chengdu 610031, China; 西南交通大学生命科学与工程学院, 四川 成都 610031; School of Life Science and Engineering, Southwest Jiaotong University, Chengdu 610031, China; 西南交通大学生命科学与工程学院, 四川 成都 610031; School of Life Science and Engineering, Southwest Jiaotong University, Chengdu 610031, China

**Keywords:** 分子印迹聚合物, 共价有机框架, 痕量残留, 诺氟沙星, molecularly imprinted polymer (MIP), covalent organic framework (COF), trace residue, norfloxacin

## Abstract

诺氟沙星(NFX)作为一种常见的喹诺酮类兽药,被广泛应用于畜牧业中,但其会残留在动物体内,进而对人体健康造成危害,为此有许多国家和组织均对NFX残留量进行了严格限制。为实现对复杂体系中痕量NFX残留的准确与可靠分析,该文制备了一种以共价有机框架(COFs)为载体的分子印迹聚合物(MIPs)。首先,在室温条件下,以金属三氟酸盐为催化剂,对苯二甲醛和3,3'-二氨基联苯为原料快速合成了“席夫碱”型共价有机框架(DP-COF)。然后将NFX、甲基丙烯酸、乙二醇二甲基丙烯酸酯与DP-COF混合,利用偶氮二异丁腈引发聚合反应,即可得到DP-COF@MIPs。整个制备过程条件温和,耗时仅5 h。采用场发射扫描电镜、傅里叶红外光谱、X射线衍射仪、BET比表面积测试仪等对其进行了表征。结果证实成功制备出了DP-COF@MIPs,该材料表面粗糙,拥有介孔范围的孔径(17.79 nm)。通过吸附实验、重复使用性实验对材料性能进行评估,结果表明该材料表观吸附容量高达41.57 mg/g,对NFX具有良好的特异性和选择性识别能力,且重复使用率令人满意。结合HPLC-UV-Vis,实现对牛奶样品中痕量NFX的检测。在3个加标水平下(0.03、0.1、0.3 mg/L),平均回收率为88.8%~92.9%,相对标准偏差小于1.7%。结果表明,该方法可以实现在复杂基质中对兽药残留高选择性、高灵敏度及准确性的检测。

## Abstract (en)

Norfloxacin (NFX) is an antibiotic that is widely used in animal husbandry. However, the presence of NFX even in trace amounts in animal-derived food may harm human health. Therefore, it is of practical significance to establish a method for monitoring NFX residues in food. Molecularly imprinted polymers (MIPs) imitate interactions established by natural receptors to selectively retain a target molecule, like antibodies or antigens do. MIPs have been widely employed in the selective recognition of specific target molecules from complex samples. Covalent organic frameworks (COFs) are a new type of organic polymer with uniform and ordered crystal structures. COFs form crystal structures by constructing organic units for ordered assembly through reversible chemical reactions. Their porous structure, regular morphology, and easy modification make COFs promising for use as excellent adsorbent carriers. Owing to these advantages of COFs, researchers have attempted to coat one MIP layer on COFs; however, the preparation methods are time-consuming and laborious, and the conditions are harsh. Hence, this study proposes a simple and rapid method for the preparation of novel MIPs with COFs as the support (DP-COF@MIPs) for the selective recognition of NFX. First, a Schiff-base COF (DP-COF) was rapidly synthesized using 3,3'-diaminobenzidine and *p*-phthalaldehyde with a metal trifluorate as a catalyst at room temperature. Subsequently, a two-step sequence was adopted as the synthesis strategy using NFX as the template, methacrylic acid as a functional monomer, and ethylene glycol dimethacrylate as a crosslinking agent. The entire synthesis was completed within 5 h under mild conditions. The material was then characterized by multiple analytical methods, including field-emission scanning electron microscopy, Fourier-transform infrared spectroscopy, X-ray diffraction, and the Brunauer-Emmett-Teller (BET) method (to determine the specific surface area). The experimental results confirmed the successful preparation of DP-COF@MIPs. The DP-COF@MIPs presented a rough and porous surface, with a pore size of approximately 17.79 nm (mesoporous structure). The properties of the material were evaluated by adsorption and regeneration experiments. The kinetic adsorption experiment showed that the DP-COF@MIPs reached adsorption equilibrium in 90 min. Two straight lines were fitted using the pseudo-second-order kinetics model based on the experimental results for thermodynamic adsorption, indicating that the adsorption process was primarily dominated by chemical adsorption. The maximum apparent adsorption capacity was 41.57 mg/g. In the selective and competitive binding test, five drugs, namely ciprofloxacin, dimetridazole, oxytetracycline, sulfadiazine, and chloramphenicol, were selected as the interferents. The experimental results showed that the DP-COF@MIPs possessed good stereoselectivity and competitive recognition ability. The regeneration of DP-COF@MIPs was evaluated by multiple cycles of adsorption-desorption experiments. The loss in the adsorption capacity of the particles was only approximately 4.7% after seven adsorption-desorption cycles. These results from the regeneration experiments show that the DP-COF@MIPs had high stability and reusability in the selective adsorption and separation of NFX. In addition, the employed method could accurately identify trace NFX in milk samples. The average recoveries were in the range of 88.8%-92.9% at three spiked levels (0.03, 0.10, and 0.30 mg/L) with relative standard deviations (RSDs) in the range of 0.6-1.7% (*n*=3). Notably, the method could successfully determine NFX at contents as low as 0.0020 mg/L in the milk sample with an average recovery of 77.6% and RSD of 6.4% (*n*=3). This concentration is one-fiftieth of the maximum residue level stipulated by the European Union (EU), and even lower than the limit of detection (LOD) of conventional high performance liquid chromatography (HPLC) methods. The above results confirm that DP-COF@MIPs can be used to determine trace NFX in actual complex samples using HPLC equipment, even when coupled to conventional UV-Vis detectors. This study offers a facile and general method for the preparation of MIPs based on COFs with selective recognition ability.

诺氟沙星(NFX)是一种氟喹诺酮类抗生素,目前广泛应用于畜牧业、养殖业。但研究发现其可能残留于动物体内,进而对人体健康造成危害^[1]^。因此许多国家和组织对肉类食品中NFX的残留进行了严格规定,如欧盟(EU)规定NFX最大残留量(MRL)为0.1 mg/kg^[2]^。针对NFX,已经发展出液相色谱-质谱联用^[3]^、毛细管电泳免疫分析法^[4]^等众多方法。但在测定之前,为消除肉类产品复杂基质的影响,在测试前均需先对样本进行前处理。

分子印迹聚合物(MIPs)是一种具有选择性的功能高分子材料。它以目标化合物为模板分子,模拟“抗原-抗体”间的分子识别作用,对模板分子表现出选择性识别能力^[5]^。近年来因其具有亲和性好、选择性高、抗干扰性强、使用寿命长等优点^[6]^,已被广泛应用于天然产物^[7]^、食品^[3]^、生物^[8]^和环境样品^[5]^中目标分子的特异性识别。共价有机框架(COFs)是一类具有均匀有序晶体结构的新型有机聚合物,由轻元素(H、B、C、N和O)组成^[9]^,是通过可逆的化学反应构建有机单元进行有序组装而形成的晶体材料^[10]^。基于其特殊结构,COFs在气体储存^[11]^、多相催化^[12]^、光电^[13,14]^、传感^[15]^和药物传递^[16]^等领域具有良好的应用前景,特别是形貌规整、表面具有多个官能团、易于修饰的特性,使COFs表现出作为MIPs载体的巨大潜力^[17,18]^。

基于COFs的显著优点,科研人员也将COFs作为载体应用于MIPs的制备^[19,20]^。其中,刘慧琳等制备了一系列针对色胺^[21]^、2,4,6-三硝基苯酚^[22]^、4-乙基愈创木酚^[23]^等的共价有机框架分子印迹聚合物复合材料(COF@MIPs)。上述COF@MIPs对目标分子表现出高特异性和高选择性,但制备过程繁琐耗时,仅COFs的合成就需在120 ℃下反应3天。在2017年,Matsumoto等^[24]^提出了一种在20 ℃下,以金属三氟酸盐为催化剂快速制备亚胺键连接的COFs。受此启发,本文针对NFX,发展了一种快速、简便制备DP-COF@MIPs的新方法,并将其成功应用于牛奶中微量NFX的检测。

## 1 实验部分

### 1.1 仪器、试剂与材料

场发射扫描电子显微镜(FE-SEM, ProX,荷兰Phenom Word公司), X射线衍射仪(XRD, PW 3040/60,荷兰PANalytical B. V.公司),傅里叶变换红外光谱仪(FT-IR, Nicolet 6700,美国Thermo Fisher公司), BET比表面积分析仪(ASAP 2020,美国Micromeritics公司),紫外-可见光谱仪(UV-Vis, UV-1800 PC,中国AOE设备有限公司),纳米粒度电位分析仪(ZEN 3600,英国Malvern公司)。HPLC仪包括LC-2030 Plus型(日本岛津公司,UV-Vis检测器,用于竞争性吸附实验)和Waters 1525型(美国 Waters公司,2998 PDA检测器,用于实际样本的测定)。

诺氟沙星(NFX)、环丙沙星(CPFX)、二甲硝咪唑(DMZ)、土霉素(OTC)、磺胺嘧啶(SDZ)和氯霉素(CAP)来自安耐吉化学品有限公司。二氧六环、3,3'-二氨基联苯胺、对苯二甲醛、1,3,5-三甲苯、三氟甲基磺酸钪(Ⅲ)、对苯二酚、二甲基亚砜(DMSO, AR, ≥99.0%)和氢氧化钠(NaOH)均来自上海阿拉丁有限公司。甲基丙烯酸(MAA)、乙二醇二甲基丙烯酸酯(EGDMA)和偶氮二异丁腈(AIBN)购自上海Adamas有限公司。甲醇、乙醇和丙酮从成都科隆化学试剂厂购得。以上试剂均为分析纯。实验全过程使用超纯水(18.2 MΩ·cm)。

牛奶样品购自四川成都市超市。

### 1.2 共价有机框架的制备

如图1所示,将43 mg 3,3'-二氨基联苯胺和54 mg对苯二甲醛分别分散在1.7 mL 1,3,5-三甲苯和0.3 mL二氧六环中,超声30 min将混合物均匀分散。随后加入3.2 mg三氟甲基磺酸钪,在氩气保护下室温放置30 min,用甲醇多次洗涤后,60 ℃真空干燥,产物记为DP-COF。

**图 1:**
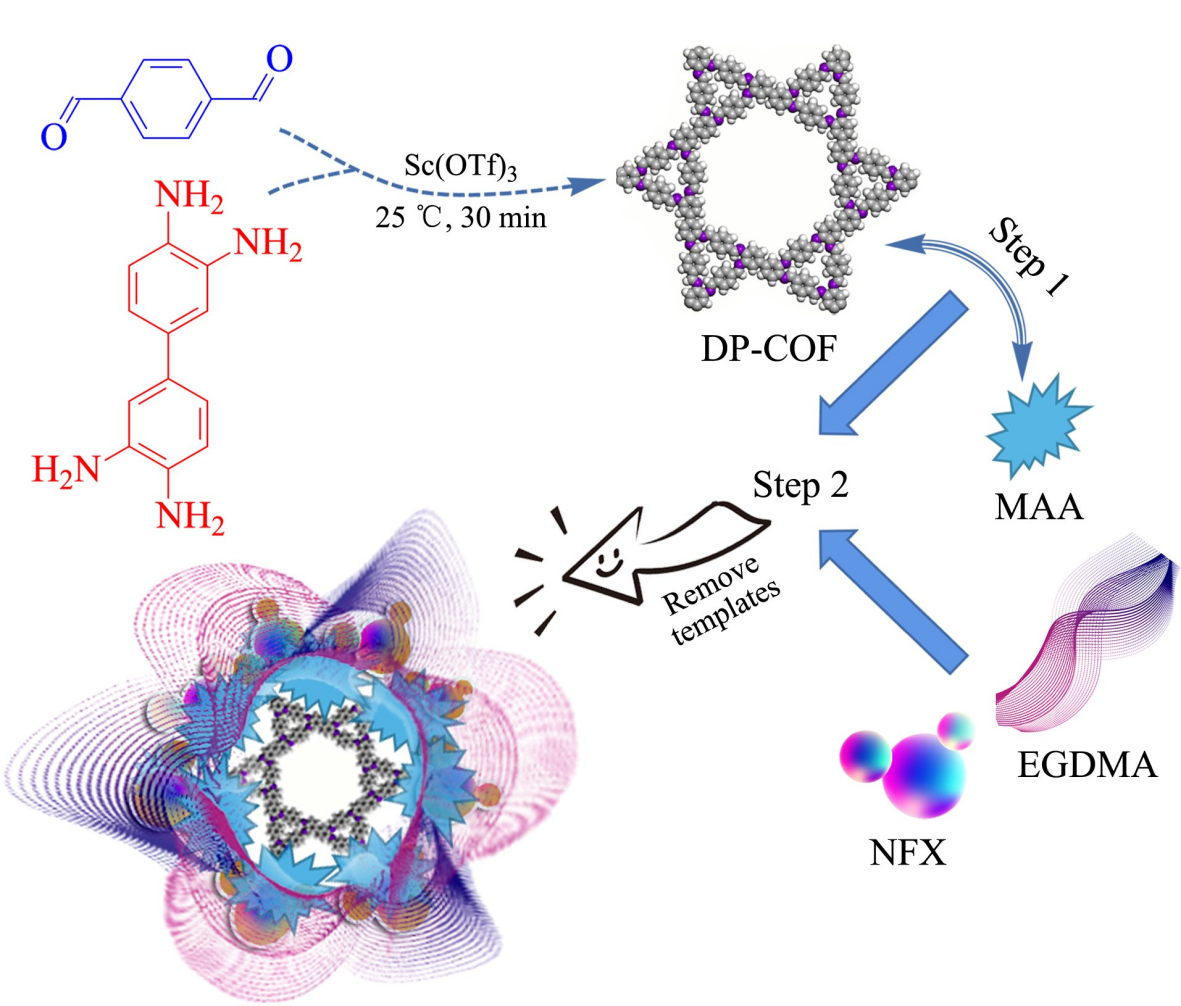
共价有机框架分子印迹聚合物(DP-COF@MIPs)的制备

### 1.3 共价有机框架分子印迹聚合物的制备

取10 mg DP-COF和0.35 mL MAA分散于3 mL DMSO中。向体系中加入4.4 mg对苯二酚,在120 ℃油浴下磁力搅拌45 min,自然冷却至室温;用0.1 mol/L NaOH溶液调至中性。加入EGDMA 2 mL, NFX 178 mg, AIBN 10 mg,丙酮5 mL,于70 ℃、磁力搅拌下反应3 h。产物经乙酸-甲醇溶液(1∶9, v/v)洗脱,以去掉结合的模板分子NFX。真空干燥后,得到共价有机框架分子印迹聚合物(DP-COF@MIPs)。

在相同的条件下(除不添加NFX)平行制备了非印迹共价有机框架聚合物(NIPs)。

### 1.4 吸附实验

等温吸附实验 将3 mg DP-COF@MIPs或DP-COF@NIPs分散在3 mL 1~300 mg/L NFX标准溶液中,孵育90 min后,8000 r/min离心3 min。使用UV-Vis分光光度计于277 nm处测定溶液中NFX的初始浓度和最终浓度。实验重复3次(下同)。

动力学吸附实验 将3 mg DP-COF@MIPs或DP-COF@NIPs分散于3 mL 10 mg/L NFX标准溶液中,按一定时间间隔(10~180 min),测定溶液上清液中NFX的浓度。

选择性吸附实验 将3 mg DP-COF@MIPs或DP-COF@NIPs分别悬浮于3 mL质量浓度均为10 mg/L的CPFX、DMZ、OTC、SDZ、CAP的标准溶液中,孵育90 min后,UV-Vis测定吸附前后溶液中NFX(277 nm)、CPFX(275 nm)、DMZ(264 nm)、OTC(354 nm)、SDZ(254 nm)和CAP(319 nm)的浓度。

竞争性吸附实验 将10 mg DP-COF@MIPs分散在3 mL NFX、CPFX、DMZ、OTC、SDZ和CAP的混合溶液中(各为10 mg/L),孵育90 min, 8000 r/min离心3 min, 3 mL甲醇-水溶液(2∶1, v/v)洗涤2次。然后用3 mL乙酸-甲醇溶液(1∶9, v/v)洗脱。使用LC-2030 Plus HPLC仪对洗脱液进行分析^[25]^: YMC-Pack C8色谱柱(150 mm×4.6 mm, 5 μm);流动相为高纯水(含0.2% NH_3_·H_2_O和0.4% H_3_PO_4_)-乙腈体系(85∶15, v/v);进样量10 μL;流速1 mL/min;柱温30 ℃;检测波长 277 nm。

### 1.5 重复使用性实验

将3 mg DP-COF@MIPs分散于3 mL 10 mg/L NFX溶液中。孵育90 min后,离心,分离出上清液。在277 nm处用UV-Vis测定溶液中NFX浓度。随后,将该DP-COF@MIPs用10 mL乙酸-甲醇溶液(1∶9, v/v)洗脱3次。然后再将DP-COF@MIPs重新放入10 mg/L的NFX溶液中,循环重复7次。

### 1.6 实际样本的测定实验

牛奶样品参照Guan等^[18]^的工作进行处理:取1 mL牛奶放入离心管中,加入3 mL乙腈和标准的NFX溶液,使NFX最终质量浓度为0、0.03、0.10或0.30 mg/L的加标样品。旋涡5 min后,于5 ℃以8000 r/min离心5 min。然后向3 mL样品中加入8 mg DP-COF@MIPs,按1.4节中竞争性吸附实验步骤对样本进行处理,但HPLC系统采用Waters 1525 HPLC, Waters Symmetry^®^ C18柱(150 mm×4.6 mm,5 μm)。除上述样本之外,还配制了一个NFX含量低至0.0020 mg/L的牛奶样本。处理过程除样本量和洗脱液为20 mL和2 mL外,其余步骤均相同。

## 2 结果与讨论

### 2.1 共价有机框架分子印迹聚合物的制备及表征

如图1所示,本实验所发展的方法与反向微乳液聚合^[26]^和一锅合成^[21]^的方法相比,条件温和,操作简单,整个合成可在5 h内完成。

图2aXRD谱中,15.0°和27.8°处的密集衍射峰分别对应于(010)和(100)面,该数据与文献报道^[27]^相符,证实了DP-COF成功制备。纳米粒度电位分析仪测定结果(图2b)表明DP-COF和DP-COF@MIPs的平均粒径分别为311.6 nm和607.0 nm。由图2c中可以明显观察到3,3'-二氨基联苯胺和对苯二甲醛分子的IR特征吸收峰(3390 cm^-1^和1695 cm^-1^,分别归于-NH_2_和-C=O);合成DP-COF后,在1610 cm^-1^处出现了新峰(-C=N伸缩振动),同时-C=O峰减弱;进一步包裹上MIPs层,FT-IR谱图变得更为复杂,同时1695 cm^-1^峰显著增强,这归因于MAA和EGDMA分子上含有的大量-C=O官能团。这证实了DP-COF和DP-COF@MIPs的成功制备。而从图3内插图中可以明显观察到DP-COF和DP-COF@MIPs及DP-COF@NIPs均为粉末,但DP-COF与DP-COF@MIPs和DP-COF@NIPs在颜色上存在明显差异,这是由于DP-COF表面包覆上聚合物层所导致的。SEM照片证实DP-COF为球形(见图3a),包覆上聚合物层后,表面变得更为粗糙(见图3b和3c);而与DP-COF@NIPs相比,DP-COF@MIPs拥有更大的孔隙。BET结果表明DP-COF@MIPs的比表面积为23.87 m^2^/g,平均孔径为17.80 nm(见图4a)。

**图 2:**
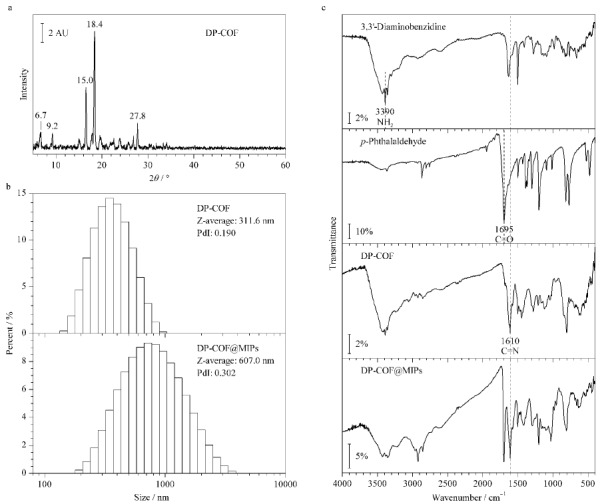
(a)共价有机框架(DP-COF)的X射线衍射图谱,(b)DP-COF、DP-COF@MIPs的粒径分布图,以及(c)3,3'-二氨基联苯胺、对苯二甲醛、DP-COF和DP-COF@MIPs的FT-IR光谱

**图 3:**
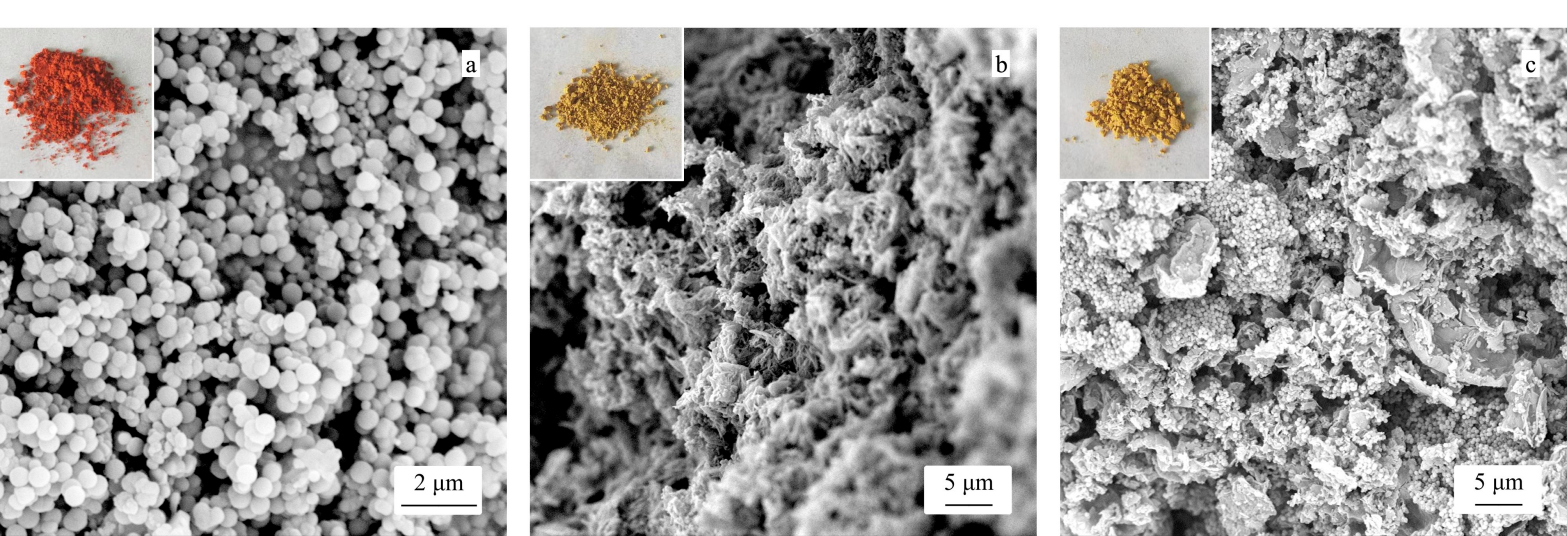
(a)DP-COF、(b)DP-COF@MIPs和(c)DP-COF@NIPs的SEM图

**图 4:**
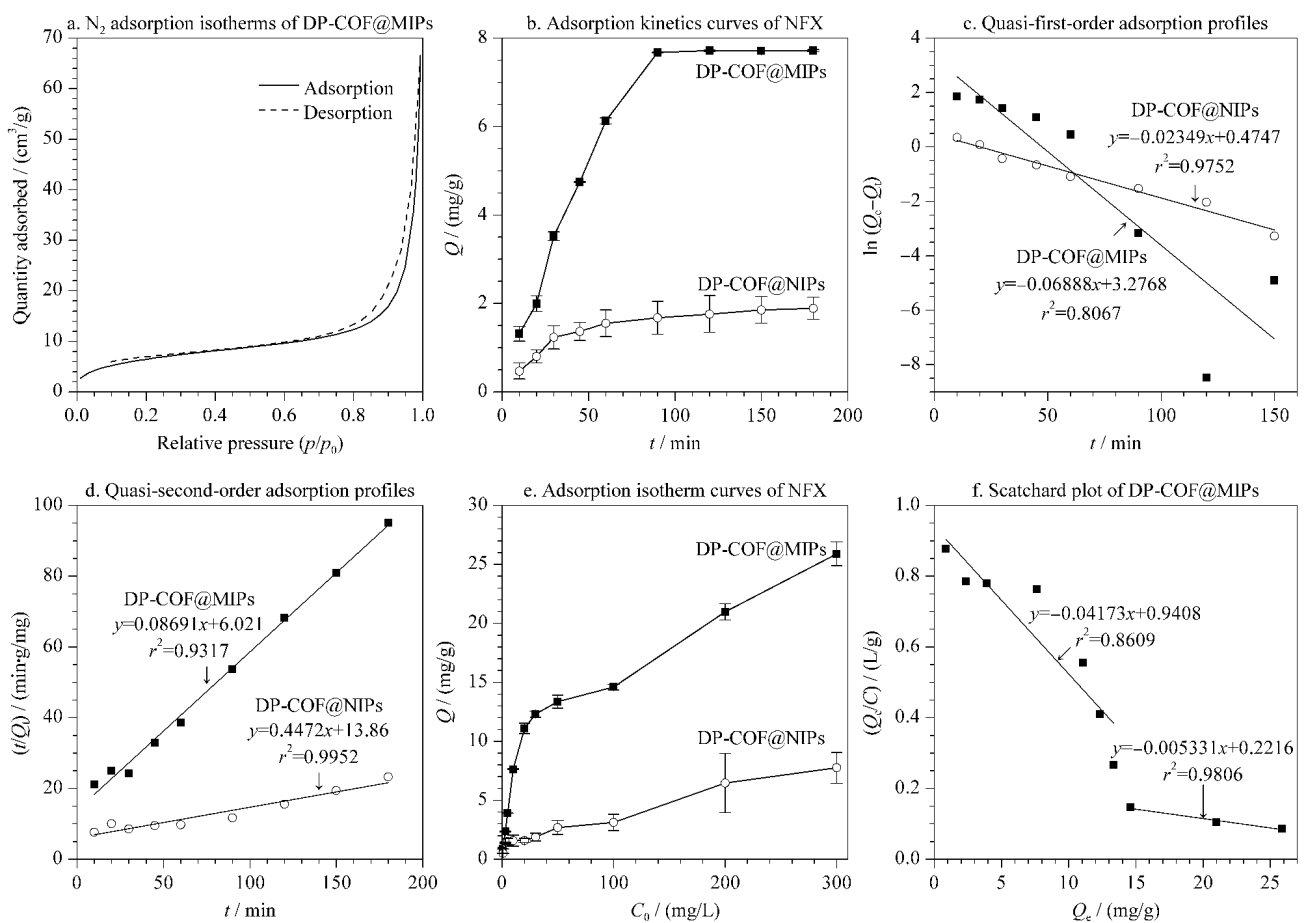
DP-COF@MIPs和DP-COF@NIPs的吸附性能评价

### 2.2 DP-COF@MIPs吸附行为研究

通过动力学吸附、等温吸附、选择性吸附和竞争性吸附实验考察了DP-COF@MIPs的吸附性能和选择性识别能力。吸附量通过公式(1)计算:


(1) $Q=\frac{\left(C_{0}-C_{\mathrm{e}}\right) \times V}{m}$

其中*Q*(mg/g)表示吸附量,*C*_0_(mg/L)为NFX的初始浓度,*C*_e_(mg/L)为NFX在溶液中的平衡浓度,*V*(L)为溶液体积,*m*(g)为DP-COF@MIPs或DP-COF@NIPs的质量。

动力学吸附实验结果如图4b所示,从图中可以看出,DP-COF@MIPs可在90 min内吸附达到平衡。DP-COF@MIPs对NFX的吸附量*Q*约为DP-COF@NIPs的3.6倍。

采用伪一级(公式(2))和伪二级(公式(3))动力学模型对动态吸附实验结果进行拟合。


(2) ln (*Q*_e_-*Q*_t_)=ln *Q*-*k*_1_*t*

(3) $\frac{t}{Q}=\frac{1}{k_{2} Q_{\mathrm{e}}^{2}}+\frac{t}{Q_{\mathrm{e}}}$

式中,*Q*_e_(mg/g)、*Q*_t_(mg/g)分别为材料在吸附平衡和时间*t*时吸附NFX的量;*k*_1_(g/(mg·min))为伪一级吸附速率常数,*k*_2_(g/(mg·min))为伪二级吸附速率常数。

对比图4c和图4d, DP-COF@MIPs和DP-COF@NIPs的动力学吸附更适合伪二级吸附模型(相关系数(*r*^2^)>0.93)。说明DP-COF@MIPs和DP-COF@NIPs的吸附过程主要受化学吸附控制。进一步利用Scatchard方程(公式(4))计算DP-COF@MIPs的表观最大吸附量。


(4) $\frac{Q_{\mathrm{e}}}{C}=\frac{Q_{\mathrm{max}}-Q_{\mathrm{e}}}{K_{\mathrm{d}}}$

其中,*Q*_e_ (mg/g)为平衡吸附量;*Q*_max_ (mg/g)为表观最大吸附量;*C* (mg/L)为平衡浓度;*K*_d_ (mg/L)为解离常数。图4e显示DP-COF@MIPs和DP-COF@NIPs的*Q*随着NFX初始浓度*C*_0_的增加而增加。在各浓度下DP-COF@MIPs对NFX的吸附量*Q*都远高于DP-COF@NIPs,证实DP-COF@MIPs对NFX良好的特异性结合能力。

如图4f所示,DP-COF@MIPs的热力学吸附数据经Scatchard方程拟合,得到了2条直线,这意味着该材料对NFX存在着高、低两种不同亲和位点。依据斜率和截距,可计算出高亲和力结合位点的*Q*_max_和*K*_d_分别为41.57 mg/g和187.62 mg/mL,低亲和力(非特异性)结合位点的*Q*_max_和*K*_d_分别为22.54 mg/g和23.96 mg/mL。值得注意的是,本工作所制备的DP-COF@MIPs表现出优异的吸附性能,其对NFX的*Q*_max_分别为核壳结构MIPs的4.83倍^[1]^,多孔粒子的1.54倍^[28]^和中空纤维的8.48倍^[29]^。

为进一步研究DP-COF@MIPs的选择性和特异性,选择我国家禽养殖业中广泛使用的CPFX、DMZ、OTC、SDZ和CAP等广谱抗菌剂作为干扰物。其中CPFX也是氟喹诺酮类抗菌剂,是NFX的结构类似物(见图5)。将DP-COF@MIPs分别处理上述6种药物,测定吸附量。由图6a可见,DP-COF@MIPs对NFX的结合量在6种药物中最高,是其结构类似物CPFX的3倍,CAP的95倍。以上结果证明DP-COF@MIPs具有出色的立体选择性。

**图 5:**
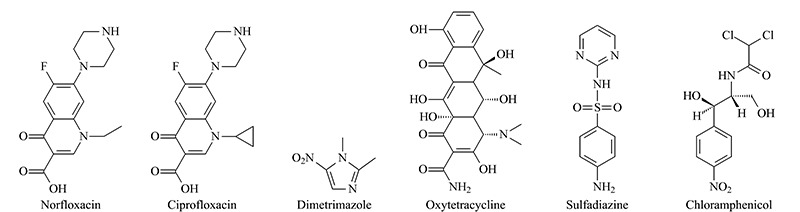
诺氟沙星和干扰物(环丙沙星、二甲硝咪唑、土霉素、磺胺嘧啶、氯霉素)的结构

**图 6:**
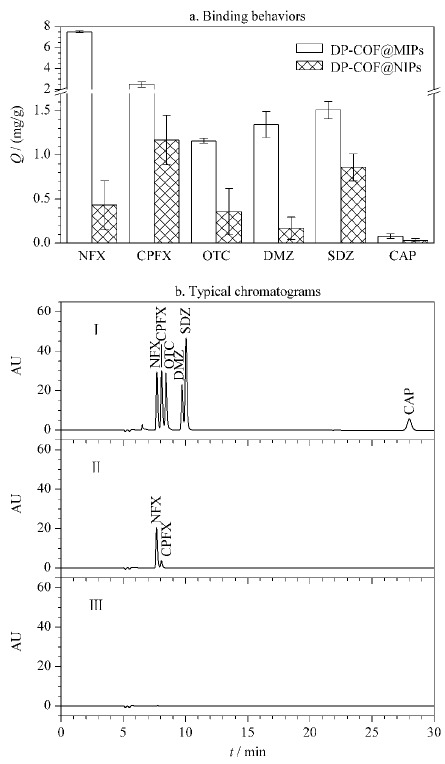
(a)DP-COF@MIPs、DP-COF@NIPs对诺氟沙星和 干扰物的吸附量对比(*n*=3)和(b)竞争性吸附实验的液相色谱图

为评价对NFX的特异性,采用DP-COF@MIPs处理含等浓度上述6种药物的标准混合液,并使用LC-2030 Plus HPLC仪对洗脱液进行分析,所得典型色谱图列于图6b。由图6b可知,所用色谱条件可以实现6种药物的完全分离。经DP-COF@MIPs处理后,在洗脱液中仅能观察到NFX及其结构类似物CPFX对应的色谱峰,且NFX峰强度远高于CPFX。与之相比,DP-COF@NIPs洗脱液中几乎观察不到任何峰。上述结果说明DP-COF@MIPs对NFX具有良好的识别能力。

### 2.3 重复使用性

对该材料的重复使用性进行了评价。连续使用7次后,DP-COF@MIPs对NFX的吸附量仍保持在初始吸附量的95.3%以上,以上结果证实该材料具有良好的重复使用性。

### 2.4 实际样本的测定结果

首先建立了Waters 1525 HPLC仪测定NFX的标准曲线,线性方程*y*=5.12×10^5^ *x*+1.15×10^4^(*r*^2^=0.999),线性范围在0.02~ 5 mg/L之间,检出限为0.005 mg/L(*S/N*=3)。然后对加标牛奶样本进行了分析。未加标牛奶、加标牛奶(0.30 mg/L)、上清液和洗脱液的典型色谱图见图7。通过对HPLC谱图对比分析,可以发现DP-COF@MIPs可以显著降低基质效应,实现牛奶中NFX的准确测定。三水平加标试验结果进一步证实了该方法的可靠性(见表1)。将所建立的方法应用于NFX含量低至0.0020 mg/L的牛奶样本的测定,平均回收率仍可达到77.6%(RSD 6.4%, *n*=3)。该浓度不仅低于所用Waters 1525型HPLC的检出限(0.005 mg/L),还仅为MRL(EU)的1/50。上述实验结果证实所制备的DP-COF@MIPs可应用于实际样本中微量NFX的高选择性富集。

**图 7:**
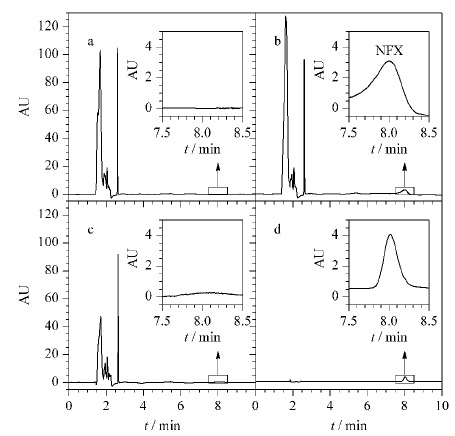
牛奶样品的HPLC图谱

**表 1:** 诺氟沙星在牛奶中3个水平下的加标回收率(*n*=3)

Added/ (mg/kg)	Measured/ (mg/kg)	Recovery/ %	RSD/ %
0.030	0.0267	88.8	1.2
0.100	0.0900	90.1	1.7
0.300	0.2786	92.9	0.6

## 3 结论

本文提出了一种在温和条件下快速制备DP-COF@MIPs的方法,所制备出的DP-COF@MIPs对目标分子表现出优异的选择性和特异性,并且具有柱容量高、重复使用性良好的特点,成功实现了实际样本中痕量NFX的检测。
